# Development of a Yellow-Seeded Stable Allohexaploid Brassica Through Inter-Generic Somatic Hybridization With a High Degree of Fertility and Resistance to *Sclerotinia sclerotiorum*

**DOI:** 10.3389/fpls.2020.575591

**Published:** 2020-11-24

**Authors:** Preetesh Kumari, Kaushal Pratap Singh, Sundip Kumar, Devendra Kumar Yadava

**Affiliations:** ^1^Indian Council of Agricultural Research (ICAR)-National Institute for Plant Biotechnology, Indian Agriculture Research Institute, New Delhi, India; ^2^Indian Council of Agricultural Research (ICAR)-Directorate of Rapeseed Mustard Research, Bharatpur, India; ^3^Molecular Cytogenetics Laboratory, Molecular Biology and Genetic Engineering, College of Basic Sciences and Humanities, Govind Ballabh Pant University of Agriculture and Technology, Pantnagar, India; ^4^Genetics Division, Indian Council of Agricultural Research (ICAR)-Indian Agriculture Research Institute, New Delhi, India

**Keywords:** inter-generic somatic hybridization, allohexaploids brassica, genomic *in situ* hybridization, seed color, *Sclerotinia sclerotiorum* resistance, *Sinapis alba*

## Abstract

The Brassica coenospeceis have treasure troves of genes that could be beneficial if introgressed into cultivated Brassicas to combat the current conditions of climate change. Introducing genetic variability through plant speciation with polyploidization is well documented, where ploidy augmentation of inter-generic allohexaploids using somatic hybridization has significantly contributed to genetic base broadening. *Sinapis alba* is a member of the Brassicaceae family that possesses valuable genes, including genes conferring resistance to *Sclerotinia sclerotiorum*, *Alternaria brassicae*, pod shattering, heat, and drought stress. This work aimed to synthesize stable allohexaploid (AABBSS) Brassica while incorporating the yellow-seed trait and resistance to *S. sclerotiorum* stem rot. The two fertile and stable allohexaploids were developed by polyethylene glycol mediated protoplast fusions between *Brassica juncea* (AABB) and *S. alba* (SS) and named as JS1 and JS2. These symmetric hybrids (2*n* = 60) were validated using morphological and molecular cytology techniques and were found to be stable over consecutive generations. The complete chromosome constitution of the three genomes was determined through genomic *in situ* hybridization of mitotic cells probed with *S. alba* genomic DNA labeled with fluorescein isothiocyanate. These two allohexaploids showed 24 hybridization signals demonstrating the presence of complete diploid chromosomes from *S. alba* and 36 chromosomes from *B. juncea*. The meiotic pollen mother cell showed 30 bivalent sets of all the 60 chromosomes and none of univalent or trivalent observed during meiosis. Moreover, the backcross progeny 1 plant revealed 12 hybridization signals out of a total of 48 chromosome counts. Proper pairing and separation were recorded at the meiotic metaphase and anaphase, which proved the stability of the allohexaploid and their backcross progeny. When screening, the allohexaploid (JS2) of *B. juncea* and *S. alba* displayed a high degree of resistance to *S. sclerotiorum* rot along with a half-yellow and half-brown (mosaic) seed coat color, while the *B. juncea* and *S. alba* allohexaplopid1 (JS1) displayed a yellow seed coat color with the same degree of resistance to *Sclerotinia* rot.

## Introduction

The Brassica coenospecies (Habard, 1976) are rich reservoirs for genes that confer resistance or tolerance to many biotic and abiotic diseases, such as Alternaria blight, Sclerotinia stem rot, insect pests, pod shattering, high temperature, and drought (Sharma et al., 2002; Li et al., 2009). At the moment, resistance gene sources for these diseases are not available within cultivated germplasms because of the established bottleneck through the continuous selection for yield traits. However, enormous genetic variations do exist in important agronomic traits, including disease resistance within the Brassicaceae family (Warwick, 1993). *Camelina sativa*, *B. desnottesii*, *Capsella bursa-pastoris*, *Diplotaxis catholica*, *D. erucoides*, *D. tenuisiliqua*, and *S. alba* are well documented for their resistance to *Alternaria brassicae* (Brun et al., 1988; Conn et al., 1988; Sharma et al., 2002), where *S. alba*, *Erucastrum cardaminoides*, *E. abyssinium*, *B. fruticulosa*, *D. tenuisiliqua* show resistance to *Sclerotinia sclerotiorum* (Li et al., 2009; Garg et al., 2010; Rana et al., 2017, 2019; Atri et al., 2019). Out of these, *S. alba* was found to be the most promising due to its resistance to major biotic and abiotic diseases that affect rapeseed and mustard, such as Alternaria blight (Kolte, 1985; Brun et al., 1987; Ripley et al., 1992; Sharma and Singh, 1992; Hansen and Earle, 1995, 1997; Sharma et al., 2002), Sclerotinia stem rot (Kolte, 1985; Li et al., 2009), blackleg, beet cyst nematode (Lelivelt et al., 1993), flea beetles (Bodnaryk and Lamb, 1991) heat, drought (Downey et al., 1975; Primard et al., 1988; Downey and Röbbelen, 1989; Brown et al., 1997; Kumari et al., 2018), and pod shattering (Downey, 1987; Chandler et al., 2005; Wang et al., 2007). Along with disease resistance properties, *S. alba* also carries quality traits, such as the yellow seed color, where yellow seed varieties are superior due to their 2–5% higher oil content relative to black seed varieties of the same genetic background (Daun and DeClercq, 1988), in addition to the high-quality and transparent oil without pigment accumulation (Slominski et al., 1994; Tang et al., 1997; Meng et al., 1998; Li et al., 2009, 2012). Therefore, breeding for yellow seed varieties is desirable by edible oil industries. Although *S. alba* is cultivated as condiment mustard in Canada with oil content less than *B. juncea’s* cultivated germplasms, the introgression of genes governing the yellow seed color and resistance to diseases into the cultivated Brassica background could help develop resistant varieties.

The allohexaploid Brassica has the potential to generate new genetic variations and introduce inter-subgenomic regions to mitigate the impact of changing climatic conditions (Gupta et al., 2016; Wei et al., 2016). The third subgenome taken from a close or wild relative could benefit oilseed crops by adding disease resistance (Chen et al., 2011) and heat tolerance genes (Kumari et al., 2018). However, the development of interspecific allohexaploids was attempted by earlier researchers by crossing of allotetraploids and diploid species followed by ovary culture, where they were treated with chromosomes doubling agents (Meng et al., 1998; Rahman, 2001; Pradhan et al., 2010; Gupta et al., 2016; Mwathi et al., 2017, 2020), but their stability was still dubious (Mwathi et al., 2019). Mason et al. (2012) used unreduced gametes by crossing all three allotetraploids of U’s triangle species, but they only obtained a single near-allohexaploid. The inter-generic fertile and stable allohexaploid development was stalled owing to pre and post-hybridization barriers between the genera in conventional breeding programs with subsequent irregular meiosis. Therefore, somatic hybridization is the best alternative approach to assemble the inter-generic genomes within a cell through allohexaploids development. The protoplast fusion of somatic cells of *B. napus* + *S. alba* (Primard et al., 1988; Lelivelt et al., 1993; Wang et al., 2005a) and *B. juncea* + *S. alba* (Gaikwad et al., 1996) were attempted earlier, but none of the stable somatic hybrids were recovered due to meiotic abnormalities or multivalent formation. Also, the back-cross progenies were derived from the somatic hybrids of *B. napus* and *S. alba* to expand their fertility (Wang et al., 2005b). Whereas in our previous attempt, we have developed the first stable and fertile somatic hybrids of *B. juncea* and *S. alba* (Australian accession) that possess brown seeds along with resistance to *Alternaria* blight and heat tolerance (Kumari et al., 2018). Of these three, two somatic hybrids have initiated flowering when day temperature rose above 30°C and efficiently set seeds up to 40°C day temperature.

In the present study, we have taken an attempt for Polyethylene glycol (PEG) mediated protoplast fusion between *B. juncea* cv. RLM-198 and an Indian accession of *S. alba* (Gene Bank Accession No. DRMR-2183) procured from the ICAR-Directorate of Rapeseed-Mustard Research, Bharatpur (India) to the genome of two different genera into a single genome by developing the fertile inter-generic allohexaploid (AABBSS) Brassica with a yellow-colored seed coat trait along with resistance to Sclerotinia stem rot. The two allohexaploids reported in the present manuscript have variabilities from earlier published hybrids for reduced plant height, early flowering, yellow, and mottle seed coat colors, and resistance to stem rot. The two allohexaploids developed during the present study acquired the mitochondria from the *B. juncea* while the two out of three somatic hybrids published earlier carried recombinant mitochondria of their parents. The genomic constitution by genomic *in situ* hybridization (GISH), genetic stability, gametic fertility, stable resistance against *S. sclerotiorum*, and the yellow seed color were investigated in subsequent self-generations of allohexaploids with their first back cross progeny. Furthermore, we studied the thickness of the palisade (pigment deposition) layer in both yellow and mottle seeded allohexaploids with their parents to uncover the histological variations inside of the yellow and mottle seed development.

## Materials and Methods

For the development of stable inter-generic allohexaploid Brassica, we have used *B. juncea* cv. RLM-198 as the cultivated germplasm with Indian *S. alba* (Gene Bank Accession No. DRMR-2183) as a donor for the yellow seed color and resistance to *S. sclerotiorum*. The *B. juncea* cv. RLM-198 was selected as a fusion parent due to its excellent regeneration potential. The three experiments included in this study were conducted using the same fusion parents.

### Somatic Fusion, Plant Regeneration, and Acclimatization

The protoplast of hypocotyl cells of *B. juncea* cv. RLM-198 and mesophyll cells of *S. alba* were isolated using the protocol of Kirti and Chopra (1990), followed by protoplast fusion, regeneration, and acclimatization according to Kirti et al. (1995). A total of five plants (clones) from each event were transferred to pots filled with soil:solarite (1:1) under net house conditions at the Indian Agriculture Research Institute (IARI), New Delhi, India and maintained till harvesting.

### Determination of Allohexaploid Organelle Constitution

Young leaves were used for the extraction and purification of genomic DNA as reported by Kirti et al. (1995), while the NanoDrop 8000 spectrophotometer (Thermo Fisher Scientific, United States) was used for DNA quantification. The mitochondrial genomic constitution was determined using polymerase chain reaction (PCR) primers specific to *orf 108* gene conferring male sterility, present in *S. alba* (Kumar et al., 2012). In primer combination (F1/R1), F1 is determined for forward and designed from the upstream region of *orf108* while R1 for a reverse primer from the coding region of atp1. In another primer combination MF/R1, the forward primer MF was designed from the DNA sequence of *M. arvensis* (Naresh et al., 2016). To determine the chloroplast genome, we tested nine plastid specific simple sequence repeats (SSR) markers, where three were given polymorphic amplicons between the parents and used to identify the chloroplast genome of somatic hybrids (Flannery et al., 2006). The PCR reaction and conditions were as described by Kumari et al. (2018), where the PCR products were resolved on a 3% agarose gel and visualized by ethidium bromide staining in a gel electrophoresis unit.

### Hybrid Morphology and Pollen Grain Viability

The fusion parents were planted with both allohexaploids in the net house at the IARI, New Delhi, India during the crop season (2015–2016). The morphological traits were recorded and compared to the parents, where the phenotypic data of five plants (clones) of both the allohexaploids of *B. juncea* and *S. alba* (JS1 and JS2) were collected at maturity during April–May, 2016. Plant height (cm), stem collar diameter, flower characteristics, main shoot length, number of siliquae on the main shoot, number of seeds per silique, and seed weight of 1,000 seeds. The same traits were recorded in the 16 lines of the first backcross progeny that was derived from both allohexaploids (JS1 and JS2) and their subsequent selfing generations across two seasons (crop season 2016–2017 and off-season 2017). The anthers were stained with 1% (w/v) aceto-carmine solution, while the pollen mother cells (PMCs) were teased with needles. Pollen grain viability was determined as the ratio of the number of stained pollen grains to the total number of pollen grains, where each microscopic field comprised 400 pollen grains that helped calculate the pollen grain viability percentage in each microscopic field.

### Chromosome Preparation and Genomic *in situ* Hybridization

The root of the subsequent selfing generation, shoot apex, and anthers of the five regenerated plants (clones) of each allohexaploid were used in the mitotic and meiotic chromosome smear preparations. In terms of the backcross progeny, three plants of each line were selected for the mitotic and meiotic preparations, where the root tips of germinating seeds or shoot leaf primordia in the mitotic studies were prefixed in 2 mM solution of 8-hydroxyquinoline for 3 h, kept in 4°C for 2 h, fixed in Carnoy’s solution I (Carnoy, 1887) with a ratio of ethyl alcohol to acetic acid of 3:1 for 72 h, transferred to 70% (v/v)ethanol, and finally stored at 4°C. The root and shoot apex were pretreated with 2% cellulase (w/v) and pectolyase (w/v) mixture for 1 h, then compressed with a 2% (w/v) solution of aceto-orcein. Similar to the mitotic studies, the floral buds were fixed in Carnoy’s solution II with a ratio of ethyl alcohol to chloroform to acetic acid of 6:3:1 for 48 h in the meiotic studies and transferred to 70% (v/v) ethyl alcohol at 4°C. The anthers were teased into a drop of 2% (w/v) acetic acid carmine solution on microscope glass slides to release the PMC, which were observed at the metaphase-I, and anaphase-I stages. Probe preparation and *in situ* hybridizations were performed as described by Kumari et al. (2020b). Post-hybridization washing was conducted at 42°C for 5 min in a 2x SSC water bath. Next, the slides were incubated in 4x SSC followed by 8x SSC, prepared in 0.2% Tween-20 (EIA grade, BioRad, United States) for 10 min, cooled down to room temperature in 2x SSC for 5 min, and then put through a dehydration series of 70, 95, and 100% alcohol. The chromosomes were stained with 2 mM 4′,6-diamidino-2-phenylindole (Sigma-Aldrich, United States) and mounted with a Vecta shield medium (Sigma-Aldrich, United States), which was visualized under a fluorescent microscope (ImagerZ2 AX10, ZEISS, Germany).

### Backcross Population Development

The plants were covered with a cloth bag to develop self-seeds that were used to grow the subsequent generation of allohexaploids. Simultaneously, to develop backcross progeny from allohexaploids, all the 10 clones of both hybrids were used as female parent and *B. juncea* cv. RLM-198 and *B. juncea* var. IJ-31 (high yielding variety) as the male parent. Each clone of allohexaploid was used for backcrossing with both *B. juncea* varieties. A total of about 30–40 flower buds for each cross were emasculated that were ready to bloom the next day and the remaining small buds were removed from the emasculated buds to avoid selfing and unwanted seed development. The fresh pollens were collected from newly opened flowers of both *B. juncea* varieties (RLM-198 and IJ-31) in the morning and pollinate the emasculated buds. The pollinated buds were covered with paper bags to avoid foreign pollen contamination. The paper bags were removed after 5–6 days and harvested all the crosses after maturity. A total of 20 back-crosses were developed and recovered approximately 22–29 siliquae from each cross. The silique developed 3–5 seeds and about 67–84 seeds harvested from each backcross. The seeds harvested from each backcross were treated as a single line in further experiments.

### Histological Studies of the Seed Coat Patterns

The physiologically matured 10 siliquae of subsequent selfing generation of each allohexaploid and their parents were fixed in formalin-acetic acid alcohol (FAA) and stored in 70% ethanol until use. Sample dehydration and clearing were performed through the alcohol xylene series, which were kept in infiltration tubes containing xylene and paraffin wax with a low melting point of 50°C under sunlight. This caused the wax to dissolve in the xylene, which was placed in an oven at 67°C for the wax to penetrate the cells and remove any traces of xylene. Next, embedding the siliquae was performed in the melting paraffin wax at 60°C, which was poured into the paper boat and infiltrated the siliqua that was placed immediately into a paper boat. Then, the boat was transferred into cold water for faster solidification of the paraffin. The solid wax cake was cut into small blocks containing a single object, where these blocks were mounted onto the block holder of the microtome to be cut into sections of 15 μ thickness and placed on clean slides with Meyer’s adhesive (Johansen, 1940). These sections were spread on the slides, dried, and stained in Delafield’s Hematoxylin, then mounted in Canada balsam, which were observed under the microscope for histological examination.

### Inoculum Preparation and Resistance Analysis of the Allohexaploids and Backcross Progeny (BC_1_) to *S. sclerotiorum*

The virulent culture of *S. sclerotiorum* was procured from Indian Type Culture Collection (ITCC), IARI, Pusa, New Delhi, India (accession: ITCC-3292). The fungal mycelium was inoculated into the potato dextrose agar medium in the Petri plates, where the *S. sclerotiorum* culture was maintained for further experiments in the cultured media at 22°C ± 1°C under alternating 12 h light and dark conditions. The developed sclerotia were harvested 12–15 days after inoculation and maintained at 4°C. The allohexaploids (JS1 and JS2) and their parents were screened with *S. sclerotiorum* during 2015–2016 to identify the resistant and susceptible plants. A total of 16 lines of BC_1_ generation of the allohexaploids, expressing high pollen grain fertility, good yield characteristics, and vigorous growth, were selected for the screening of stem rot disease along with both allohexaploids and their parents during the rabi season in 2017–2018 (season-I) at the IARI agriculture farm, Pusa Campus, New Delhi, India and rescreened during the offseason in 2018 (season-II, June to September) at the IARI Regional Station, Katrain, Kullu, Himachal Pradesh, India. The plants of BC_1_ line, both allohexaploids, and their parents were sown in a randomized block design with three replications. Five plants from each replication were selected for pathogen inoculation after the initiation of flowering, where the selected plants were inoculated with 6 mm discs of actively growing virulent pathogens on agar plates, at just above the first node on a hand scratched point, and tied with parafilm (Garg et al., 2010). The disease progress was monitored regularly, and the final stem lesion sizes were recorded after 21 days of inoculation. The plants were categorized according to lesion size in terms of immune (no sign of disease), highly resistant (0–2.0 cm), resistant (2.1–4.0 cm), moderately resistant (4.1–6.0 cm), tolerant (6.1–8.0 cm), susceptible (8.1–10.0 cm), and highly susceptible (more than 10.0 cm).

## Results

To the development of the allohexaploids for introgression Sclerotinia stem rot resistance along with yellow seed coat color, the PEG mediated somatic hybridization was done between *B. juncea* cv. RLM-198 and *S. alba*. Hybridity was evident from morphological features, chromosomal constitutions by Genomic *in situ* hybridizations (GISH), and organelle constitutions determined through molecular analysis. Disease resistance was confirmed by screening using virulent isolate of *S. sclerotiorum* of the two allohexaploids and their first backcrossed progeny and parental checks in field conditions.

### Development of Stable Allohexaploids by the Protoplast Fusion of *B. juncea* and *S. alba*

**FIGURE 1:**
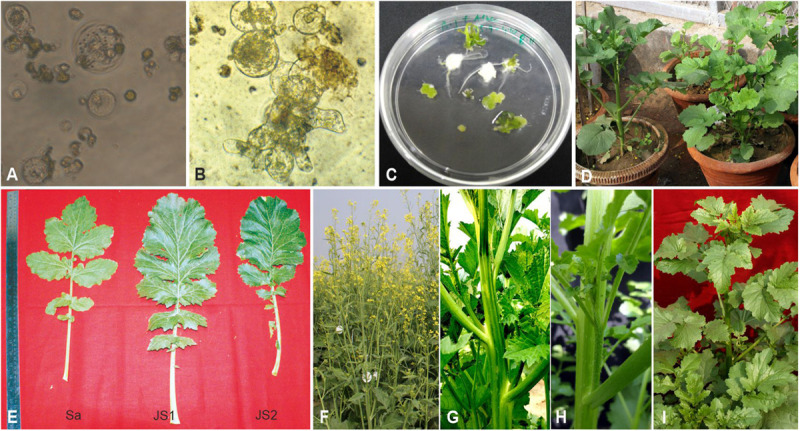
Protoplast fusion between *B. juncea* and *S. alba*. **(A)** Protoplast fusion product after 48 h of fusion. **(B)** Subsequent cell division and cell wall formation. **(C)** Initiation of shoot regeneration from calli after 35 days of fusion. **(D)** Allohexaploids established in pots. **(E)** Morphological variation in leaf. **(F)** Plant morphology of *B. juncea* cv. RLM-198. **(G)** Stem morphology of JS1 showing angular stem with prominent trichomes. **(H)** Stem morphology of JS2 showing prominent trichomes. **(I)** Plant morphology of *S. alba*.

Morphologically distinct protoplasts were used for protoplast fusion, i.e., hypocotyl protoplasts of dark-grown seedlings of *B. juncea* were colorless, while the mesophyll protoplasts of *S. alba* were visibly green, therefore, the hetero-fusion products were easily differentiated. Protoplasts of both parents were kept unfused as a control. The hybrid fusion products were detected using the green chloroplasts that were grouped at the center 2 days after fusion (Figure 1A). Cell wall synthesis began 56 h after fusion when the protoplasts lost their spherical shape (Figure 1B). Cell division was initiated 72 h after fusion with consecutive cell divisions observed up to 7 days after fusion. The division also began after 72 h in the protoplast of *B. juncea* RLM-198, which was kept unfused. However, in the *S. alba* control, neither the protoplast division nor the cell wall formation was observed. The division rate was inconsistent in the three experiments and ranged between 20 and 50%. The fusion product developed in the form of micro-colonies 20 days after fusion. A total of 954 microcalli were obtained from the three experiments, where a total of 346 calli were transferred into the regeneration medium that was standardized for *B. juncea* cv. RLM-198 regeneration and the pH was maintained at 5.8 after the addition of plant hormones and obtain 56 shoots on the 5th week of protoplast culture (Figure 1C). All 56 shoots were treated as 56 independent events. The majority of these shoots readily developed roots on the half-strength Murashige and Skoog medium. A total of 54 plantlets showed morphological resemblance to *B. juncea* more than *S. alba*, while only two plantlets named JS1 and JS2 after transplantation in the net house showed an intermediate morphology between their parents, which were transferred into pots and grown under the net house (Figure 1D). During the early growth stages, we found that JS1 and JS2 showed more resemblance to *S. alba*, but after a few weeks, their morphology became an intermediate of the two parents in terms of leaf shape, leaf size, leaf texture, stem, size, stem shape, presence of prominent trichomes on leaves and stem, the emergence of flower buds, flower shape and size, anther, siliquae shape and size, and beak shape.

The stem of *B. juncea* was smooth, light green, and without trichomes (Figure 1F). The stems of JS1 and JS2 were angular, branched, and covered with dense trichomes (Figures 1G,H). Moreover, the stems of *S. alba* also found rough, angular with dense trichomes (Figure 1I). The leaf and flower bud margins were similar to *S. alba* with JS1 and JS2 attaining an average height of 216 and 184 cm, respectively. The delayed flowering was recorded in the allohexaploids relative to *B. juncea*, where the leaves were considerably larger, thicker, more cuticular, deeper green in color, had more irregular pinnately lobed serrate margins, and was more covered with dense trichomes on both surfaces (Figure 1E). The inflorescence of JS1 showed an *S. alba* like condense pattern, while JS2 had an intermediate pattern of the parents. JS1 and JS2 flower sizes were larger and intermediate relative to the parents’ flowers, respectively. The stigma was larger than that of the parents with hairy projections on the style, while the stamens of allohexaploids were more prominent than in the parents (Table 1). The pollen grain viability in both allohexaploids was between 91 and 93% (Figure 4A) with female fertility remaining high as revealed by their seed setting. The siliquae of the allohexaploids were found to be intermediate in size relative to their parents with an increased number of seeds (3–5 seeds/silique) compared to *S. alba*. These allohexaploids produced viable seeds when selfing and backcrossing with *B. juncea*, which were found to be intermediate in size relative to the parents. However, the weight of 1,000 JS1 and JS2 seeds was higher and less than that of *B. juncea* and *S. alba*, respectively (Table 1). Allohexaploid JS1 produced yellow seeds (Figure 4B) that were similar to *S. alba*, while JS2 interestingly produced half yellow half brown (mosaic) colored seeds (Figure 4C). To the best of our knowledge, this was the first instance of obtaining mosaic and yellow seeds from somatic hybridization of *S. alba* and *B. juncea*, where the seeds colors were stable over the generations in both allohexaploids.

**TABLE 1:** Comparative morphological characterization of *B. juncea* cv. RLM-198, *S. alba*, JS1, and JS2.

**S. nos.**	**Parameters**	***B. juncea* Mean** ± **SD**	***S. alba* Mean** ± **SD**	**JS1 Mean** ± **SD**	**JS2 Mean** ± **SD**
1.	Plant height (cm)	156.66 ± 2.60	163.20 ± 0.986	216.25 ± 0.84	181.33 ± 1.24
2.	Stem diameter (cm)	13.12 ± 1.16	15.59 ± 3.12	25.12 ± 1.56	23.60 ± 1.64
3.	Leaf length (cm)	21.34 ± 1.56	34.97 ± 1.67	61.56 ± 3.13	54.24 ± 1.87
4.	Leaf width (cm)	13.97 ± 1.07	19.20 ± 1.02	29.50 ± 1.10	26.90 ± 0.80
5.	Days of flowering	67	110	86	90
6.	No. of primary branches	8.97 ± 1.26	12.97 ± 4.95	16.5 ± 1.70	16.33 ± 2.52
7.	No. of secondary branches	12.30 ± 0.84	64.80 ± 9.38	73.50 ± 1.27	67 ± 1.22
8.	Length of petals (mm)	11.00 ± 0.42	12.75 ± 0.21	16.00 ± 0.87	14.75 ± 0.80
9.	Width of petals (mm)	6.25 ± 0.45	8.20 ± 0.16	9.20 ± 0.68	8.70 ± 0.20
10.	Length of stamens of lateral whorl (mm)	6.15 ± 0.53	7.50 ± 0.41	8.15 ± 0.58	7.95 ± 0.96
11.	Length of stamen of median whorl (mm)	9.25 ± 0.23	10.80 ± 0.64	12.10 ± 0.36	11.15 ± 0.81
12.	Length of carpel (mm)	7.65 ± 0.96	10.20 ± 0.12	13.20 ± 0.59	12.10 ± 0.91
13.	Length of main shoot (cm)	39.60 ± 0.693	25.80 ± 3.89	41 ± 1.94	49.33 ± 0.69
14.	No. of siliquae on main shoot	44.80 ± 0.46	39.0 ± 0.647	51.25 ± 0.93	47.67 ± 0.84
15.	Silique length (cm)	5.4 ± 0.18	2.9 ± 0.16	3.89 ± 0.45	3.33 ± 0.67
16.	Silique beak length (cm)	0.59 ± 0.09	1.27 ± 0.89	1.1 ± 0.25	0.93 ± 0.27
17.	No. of seeds/silique	12.60 ± 0.21	4.6 ± 0.94	9.68 ± 1.97	9.30 ± 1.83
18.	1,000 seed weight (gm)	4.20 ± 0.46	2.90 ± 0.98	4.10 ± 0.19	3.12 ± 2.41

### Molecular Analysis of the Organelle Genome Constitution

**FIGURE 2:**
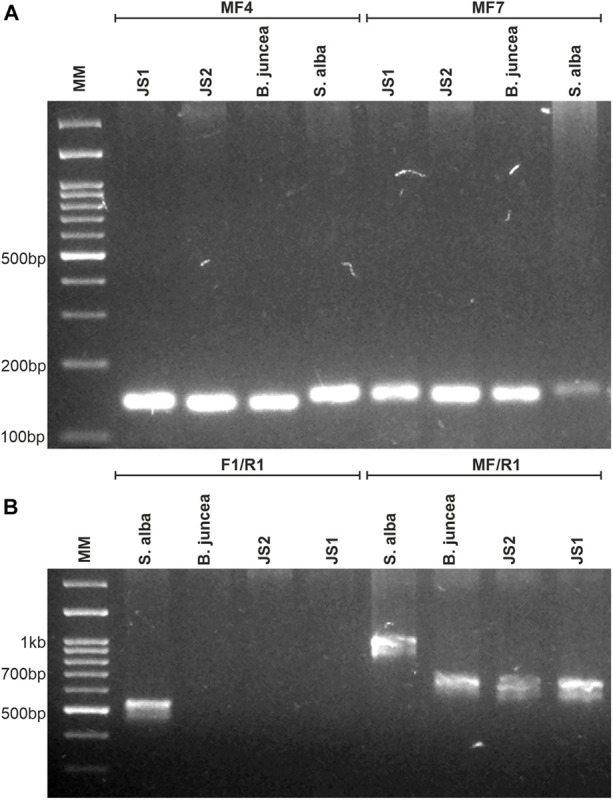
Molecular analysis for determination of organelles constitution of allohexaploids and their parents (*B. juncea* and *S. alba*). **(A)** PCR amplification patterns of MF4 and MF7 markers specific for chloroplast genome, showing inheritance of *B. juncea* chloroplast in both allohexaploids. **(B)** PCR amplification obtained in both allohexaploids and their parents using orf-108 primers specific for mitochondria, the F1/R1 and MF/R1 primer combinations showing *B. juncea* type mitochondria in allohexaploids (JS1 and JS2).

Out of the nine tested chloroplast genome-specific SSR markers, only three markers showed polymorphism between the parents that were used to determine the chloroplast constitutions of the allohexaploids. JS1 and JS2 provided amplicons specific to *B. juncea* and confirmed the acquisitions of chloroplast from the *B. juncea* in both allohexaploids (Figure 2A). Similarly, the mitochondrial constitution of both allohexaploids was also found as similar to *B. juncea*, which was determined through PCR using primers that corresponded to *orf108* upstream of the *atp*A gene. The primer combination of F1/R1 gave amplicon ∼530 bp in *S. alba* while absent in *B. juncea* and both allohexaploids. Another primer combination MF/R1 produced polymorphic amplicons between the parents for 1 kb and ∼680 bp in *S. alba* and *B. juncea*, respectively. However, both allohexaploids produced similar amplicons of *B. juncea* (Figure 2B and Table 2).

**TABLE 2:** Details of PCR primer pairs used in the study.

**S. nos.**	**Primer name**		**Sequence**	**Amplicon size (bp)**
1.	MF-4	F R	CGGATCTATTATGACATATCC GAAATATGAATACACTAGATTAGG	155
2.	MF-7	F R	CGGCAGGAGTCATTGGTTCAAA GATTTTGTAACTAGCTGACG	171
3.	F1/R1	F R	TACTCCTAGAGGCTTGACGG GCAGCTCTGGGAGATAATTCC	530
4.	MF/R1	F R	CTTGCAGACCTACTCGGAAC GCAGCTCTGGGAGATAATTCC	1,000

### Cytological Studies Using Genomic *in situ* Hybridization

**FIGURE 3:**
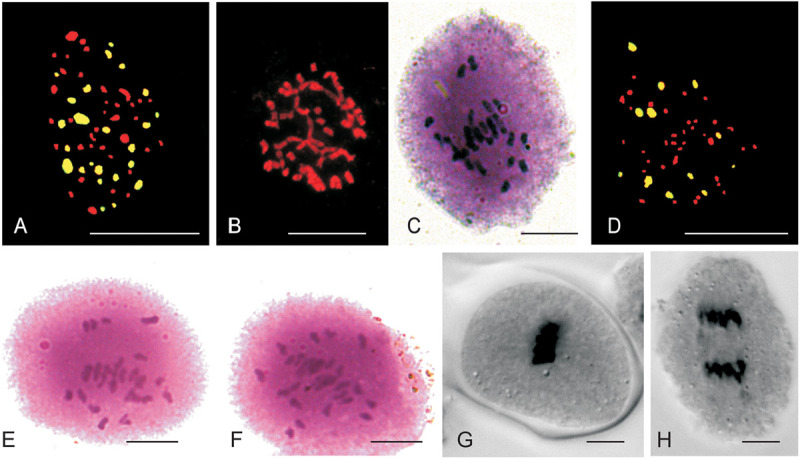
Mitotic and Meiotic studies of allohexaploids and BC_1_ progeny. **(A)** 24 FITC labeled *S. alba* (green) chromosomes and 36 *B. juncea* chromosomes (red) of JS1. **(B)** Somatic chromosomes of JS2 (2*n* = 60). **(C)** Thirty bivalent observe in PMCs of JS1. **(D)** 12 FITC labeled *S. alba* chromosomes in BC_1_ of JS2 (AABBS = 48) with green color and 36 chromosomes of *B. juncea* in red color. **(E,F)** 24 bivalents seen in JS1 and JS2 at diakinesis of meiosis-I, respectively. **(G)** Meiotic metaphase of BC_1_ (JS1). **(H)** Meiotic anaphase-I of BC_1_ (JS1) (Scale bar 10 μm).

The mitotic chromosome count of JS1 and JS2 revealed that both allohexaploids carried 60 chromosomes reflecting a cumulative chromosome count of *B. juncea* (2*n* = 36) and *S. alba* (2*n* = 24) (Figures 3A,B). The *S. alba* chromosome constitution was detected by 24 strong hybridization signals using genomic *in situ* hybridization (GISH) with a fluorescein isothiocyanate labeled genome-specific probe of *S. alba* (Figure 3A). This chromosomal stability was also been observed in subsequent generations, where a total of 30 bivalents were noted at meiosis-I in the PMCs of JS1 (Figure 3C) and maintained a high degree of fertility. The BC_1_ progeny of hybrids using the *B. juncea* backcrossing was found to be highly fertile in terms of the male and female gametes. This fertility was maintained with a haploid set (*n* = 12, S) of the *S. alba* chromosomes along with a diploid set of *B. juncea* chromosomes (2*n* = 36, AABB), a total 48 chromosomes were evident from mitosis of first backcross (BC_1_) generation (Figure 3D). The normal 24 bivalents were observed at diakinesis of meiosis-I in both allohexaploid (JS1 and JS2) (Figures 3E,F). All 48 chromosomes were observed at the equator as normal bivalents at metaphase-I and no laggard chromosome evident (Figure 3G) and equal distribution toward the poles at anaphase-I was observed (Figure 3H).

### Histology of the Seed Coat Developmental Pattern

**FIGURE 4:**
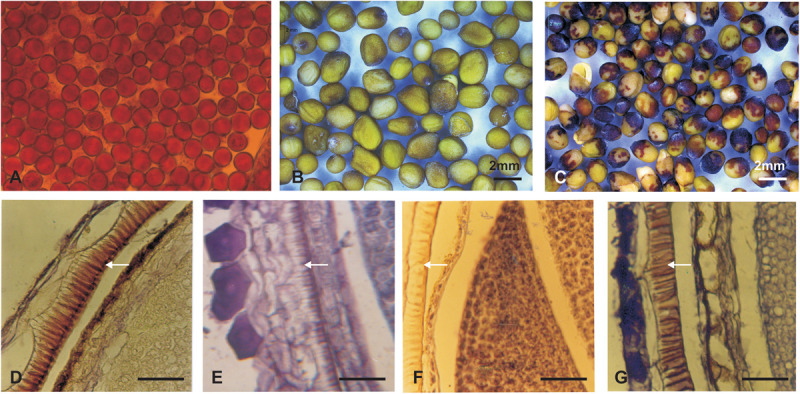
Pollen viability and seed coat colors (upper panel). **(A)** Pollen grain viability test of JS2. **(B)** Yellow color seeds of JS1. **(C)** Yellow–brown color seeds of allohexaploid JS2. **(D–G)** Mature silique histology showing depositions in palisade layer of the seed coat (lower panel). **(D)** *B. juncea*. **(E)** *S. alba*. **(F)** Allohexaploid JS1. **(G)** Allohexaploid JS2 (Scale bar 25 μm).

The histological study of the seed coat revealed that the pigment distribution was mainly observed in the palisade layer, where the highest pigmentation was observed in *B. juncea* (Figure 4D), moreover *S. alba* and yellow seeded allohexaploid (JS1) showed very low pigmentations in their palisade layer (Figures 4E,F). The mottled seed allohexaploid (JS2) exhibited nearly *B. juncea* like pigment deposition in the palisade layer (Figure 4G).

### Screening for Sclerotinia Stem Rot Resistance in the Allohexaploids (JS1 and JS2) and Their Backcross Progeny

**FIGURE 5:**
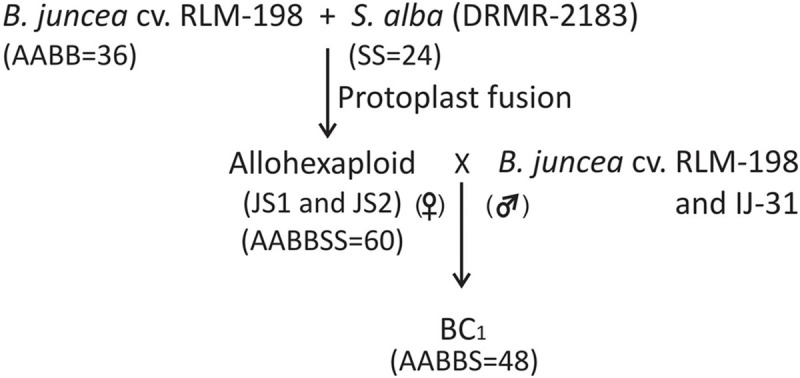
Flow chart diagram showing the development of allohexaploids and their first backcross progeny (BC_1_).

**FIGURE 6:**
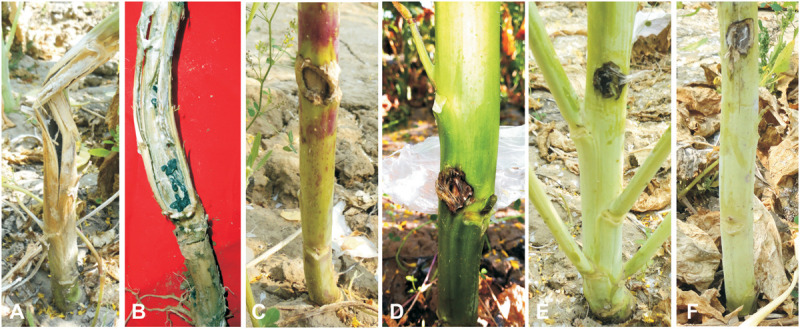
Screening against *Sclerotinia sclerotiorum*. **(A,B)** *Sclerotinia* rot symptoms on *B. juncea* stems as a susceptible check with sclerotia formation. **(C)** Inoculated plant of *S. alba* as a resistant check. **(D)** Allohexaploid JS1. **(E)** Allohexaploid JS2. **(F)** BC_1_ progeny of Allohexaploid JS1.

**FIGURE 7:**
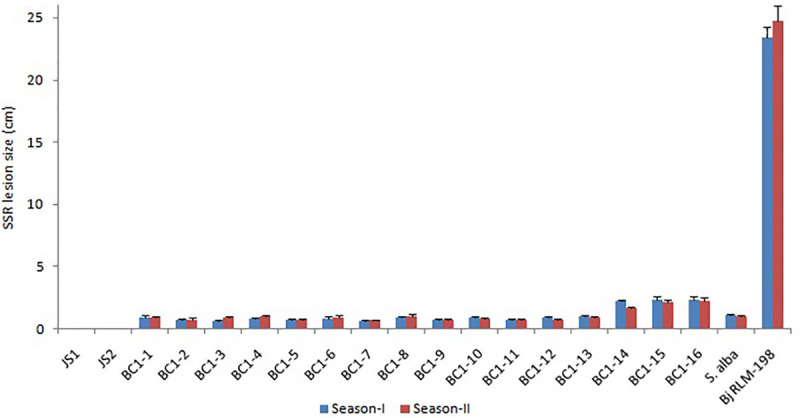
The Sclerotinia stem rot screening results of allohexaploids and their BC_1_ generation with parental checks during two crop seasons (Bar-StDv).

The allohexaploids (JS1 and JS2) screened for stem rot resistance during 2015–2016 showed a complete immune response against the pathogen as it was unable to grow on the stem surface. However, *B. juncea* was found highly susceptible to the disease as the complete stem was rotted by the pathogen, while *S. alba* showed very limited growth on the stem surface, but it did not develop any characteristic disease symptom. In terms of the BC_1_ generation of both allohexaploids, a total of 16 lines were selected for disease screening experiments based on their pollen grain fertility (> 90%), good yield, and vigor along with both the allohexaploids (JS1 and JS2) and their parents (Figure 5). The stem rot pathogen produced typical disease symptoms on the susceptible parent *B. juncea* cv. RLM-198 with lesion sizes between 22.4–24.9 cm and 23.1–26.6 cm during season-I and season-II, respectively. The complete collar area of the stem was rotten (Figure 6A) and produced sclerotia within it at advanced disease stages (Figure 6B). The pathogen grew on the stem surface of *S. alba* without developing any characteristic rotten disease symptoms (Figure 6C). However, lesion sizes were recorded between 1.0–1.2 cm and 1.0–1.1 cm during both experimental seasons on the upper epidermis only. The stems of both hybrids grew vigorously and did not show any sign of disease at the inoculation point till harvesting (Figures 6D,E). The results of season-I showed that 13 lines of BC_1_ were of the highly resistant category as the disease lesion size was recorded between 0.6 and 1.1 cm (mean = 0.8 cm) (Figure 6F). Another 3 BC_1_ lines were found to be resistant to the disease as their lesion sizes ranged between 2.1 and 2.8 cm (mean = 2.3 cm). As expected, it was found that the BC_1_ generation showed highly resistant responses to the stem rot pathogen compared to the susceptible *B. juncea* parent. In season-II, the 14 and 2 lines of BC_1_ progeny were found highly resistant to the disease with mean lesion sizes of 0.9 and 2.2 cm, respectively. These results indicated that BC_1_ generation was consistently resistant to the disease and had very little variation in mean lesion sizes in both years (Figure 7).

## Discussion

Inter-generic allohexaploid Brassicas are a potential source for gene introgression conferring resistance against major biotic and abiotic stresses of cultivated Brassica (Primard et al., 1988; Lelivelt et al., 1993; Hansen and Earle, 1997; Wang et al., 2005a, 2006; Kumari et al., 2018). The somatic hybridization is an approach to overcome problems associated with inter-generic incompatibility barriers and assists the inter-generic hybridization (Kirti et al., 1995).

There were two major impediments in successful introgression of the disease resistance from coenospecies to crop Brassicas by somatic hybridization i.e., poor regeneration frequency of fusion products and meiotic abnormalities in allohexaploids. A very low regeneration frequency (0.1 and 0.18–0.81%) was recorded after the protoplast fusion of *B. napus* and *B. oleracea* with *S. alba* by Primard et al. (1988) and Hansen and Earle (1997), respectively. Although, we have successfully overcome the limited regeneration by standardization of the regeneration medium and recorded a 5.9% regeneration frequency during our earlier study (Kumari et al., 2018). Further, significant improvement was observed with a 16.2% regeneration frequency from the protoplast fusion of *S. alba* and *B. juncea* in present experiments. The high regeneration frequency confirmed the advantage of standardization of pH after adding hormones into the regeneration medium. The lower male and female fertility were another constrain to move toward inter-generic allohexaploid development through somatic hybridization (Kirti et al., 1992, 1995). The multivalent formations were frequently observed at the meiosis of inter-generic allohexaploids, resulting in low male and female fertility (Primard et al., 1988; Wang et al., 2005b, 2006). Besides this, the male sterility has been recorded even after the predominant pairing at meiosis in allohexaploids of *B. juncea* + *S. alba* and resultant seed setting failed during self-pollination. Whereas, satisfactory seed setting was recorded only after backcrossing with *B. juncea* pollens and using allohexaploids as a female parent (Gaikwad et al., 1996). Nonetheless, we have observed a high male and female fertility after normal pairing and formation of 30 bivalents at diakinesis in three allohexaploids of *B. juncea* + *S. alba* (Kumari et al., 2018). We have also obtained high degree male and female fertility in the present two allohexaploids developed by using the Indian accession of *S. alba* (DRMR-2183) instead of the Australian accession which was used in our previous study. Gaikwad et al. (1996) confirmed that the allohexaploids of *B. juncea* and *S. alba* acquired chloroplast constitution from the *S. alba*. However, in the present study both allohexaploids (JS1 and JS2) acquired their chloroplasts from *B. juncea*, and the same chloroplast constitution was observed in our earlier three stable hybrids (Kumari et al., 2018). Thus, there might be a possibility that *S. alba* chloroplast causing male sterility in allohexaploids even after predominant pairing during meiosis. We have also found *B. juncea* type mitochondria in both allohexaploid (JS1 and JS2). Whereas, recombinant mitochondrial constitutions have been evident in our previous two allohexaploids (H1 and H3) out of three while H2 acquired their mitochondria from *B. juncea* (Kumari et al., 2018). The recombinant mitochondrial constitutions of their parents were reported frequently in inter-generic somatic hybrids (Kirti et al., 1992, Kirti et al., 1995; Lelivelt et al., 1993).

The inter-generic allohexaploids developed through somatic hybridization were often reported to possess intermediate morphology of parents presumably due to the presence of cumulative chromosome numbers of their parents (Kirti et al., 1992; Gaikwad et al., 1996; Wang et al., 2005a, 2006). Similarly, we have found intermediate morphology at some growth stage during our previous study like time to flower, while for other traits the allohexaploids were superior to their parents such as the presence of apparent trichomes on stems and leaves, vigorous plant growth, and branching patterns. Nonetheless, the seed color of all three allohexaploids (H1, H2, and H3) was brown similar to *B. juncea* parent (Kumari et al., 2018). However, in the present study, the intermediate phenotypes were observed such as seed color in JS2 (Half yellow and half brown), flowering initiation time, siliquae length, siliquae beak length, number of seeds per silique, and seed weight in both allohexaploids JS1 and JS2. Whereas other traits resembled their resistant donor parent (*S. alba*) such as the presence of trichomes on stems and leaves, yellow seed color of JS1. The plant height in both the allohexaploids was found more than their parents but much smaller than the earlier brown seeded (H1 and H2) allohexaploids which were also reported tolerant to high temperature (up to 40°C) during seed set.

Wang et al. (2005b) have observed inter-genomic association in first backcrossed (BC_1_) progeny of *B. napus* + *S. alba* allohexaploids with the sequidiploid chromosomal constitution (AACCS = 50) and observed 4 trivalent during meiosis. Similarly, we have found a haploid set of *S. alba* chromosomes (S = 12) in sequidiploid chromosome constitution (AABBS = 48) in the first backcross generation and observed normal pairing between the 48 chromosomes. We have observed 24 bivalents at the meiosis-I of BC_1_ and no univalent, trivalent and laggard chromosome was seen at meiotic metaphase. However, any precise study regarding autosyndesis in *S. alba* or allosyndesis within *B. juncea* and *S. alba* genomes are not available, some presumption comes from the present study and is supported by Gaikwad et al. (1996). Considering the phylogeny and the cytological literature and BLAST the *B. juncea* sequences with the draft genome of *S. alba* both genomes share 78% similar sequences. Therefore the chances for the inter-genomic association are greater, Although, further validation is required to confirm this point of view.

Yellow and half yellow and half brown (mottle) seed color has been recorded for the first time in allohexaploids developed through somatic hybridization and their BC_1_ generation. So far, the yellow seed color is also not recorded in somatic hybrids. However, the yellow seed color introgression has been observed after four rounds of successive backcrossing (BC_4_F_4_) in *B. napus* + *S. alba* somatic hybrids in addition to Sclerotinia stem rot resistance (Li et al., 2009). Although, the yellow seed varieties are available within *B. juncea* but preliminary reports have mentioned that the distinct testa color of *S. alba* differed from other yellow seeded germplasms available in the crop Brassicas. To the best of our knowledge, the introgression of the yellow seed color was not attempted from *S. alba* to *B. juncea* so far. Li et al. (2012) have identified a characteristic *S. alba* specific 288 bp amplicon responsible for yellow seed color in BC_4_F_4_ generation of *B. napus* + *S. alba* somatic hybrids. This amplicon sequence was not matched with *B. napus* genome assembly available in the National Center for Biotechnology Information. Thus, they have suggested that it might be a part of the *S. alba*-specific gene sequence. We have used this 288 bp sequence DNA sequence in the Basic Local Alignment Search Tool Nucleotide (BLASTN) with the *S. alba* draft genome assembly (Kumari et al., 2020a) and found three copies of approximately similar sequences in *S. alba* genome. Apart from this, we have studied the histology of physiologically mature seeds of both allohexaploids (yellow and mottled seeded). The palisade thickening of yellow seed was similar to *S. alba*, while the mottle seeds revealed that their palisade layer thickening closer to *B. juncea* which was agreed with the findings of Li et al. (2009) for yellow seeded progenies. However, further validation is required to identify the genes responsible for yellow and mottle seed colors. The subsequent self-generations and backcross progenies of the *B. juncea* + *S. alba* allohexaploids were found to be highly stable and resistant to Sclerotinia stem rot disease.

A total of 16 lines from BC_1_ derived from both allohexaploids with their parent used as checks were screened in the field by stem inoculation and gave an immune response to high degree resistance against a virulent isolate of *S. sclerotiorum*. A similar resistance response was recorded in BC_4_F_4_ progeny of *B. napus* and *S. alba* somatic hybrids for Sclerotinia stem rot resistance by *in vitro* leaf inoculation and found higher resistance than the known resistant rapeseed variety “Zhongshuang 9” (Li et al., 2009). Although, *S. sclerotiorum* pathogen is attacked on the stem and causing severe yield loss but no significant correlation was noticed between leaf and stem resistance. Taylor et al. (2015) concluded that the resistance in these two different tissues (stem and leaves) might be controlled by different genes. Similar findings were reported in ILs of *B. juncea-B. fruticulosa*, where highly resistant plants with lesion sizes less than 2.5 cm were recorded (Rana et al., 2017). Atri et al. (2019) identified 13 loci responsible for *S. sclerotiorum* resistance on seven different *B. juncea* chromosomes that were used in the set of *B. juncea-B. fruticulosa* ILs. In our study, the allohexaploids (JS1 and JS2) were found highly resistant to the *S. sclerotiorum* during three seasons (2015–2016 and 2017–2018 crop).

Thus, the stable allohexaploids of *B. juncea* + *S. alba* and their backcross progeny are developed in the present study, can provide the genetic resources for novel agronomically important traits and provides new insights into the genetic inheritance of traits such as the resistance to Sclerotinia stem rot and yellow seed color. Breeding for the introgression of these traits into another Brassica crop could be achieved as these allohexaploids are confirmed their cross ability with diploid and amphidiploids of cultivated Brassicas.

## Data Availability Statement

The original contributions presented in the study are included in the article/Supplementary Material, further inquiries can be directed to the corresponding author.

## Author Contributions

PK designed, conducted lab and field experiments, the data collection, preparation of cytological smears and GISH, and drafted, edited, and finalized the manuscript. KS contributed to seed coat developmental histology, conducted field studies, *Sclerotinia sclerotiorum* culture maintenance, screening stem rot resistance, the data collection and analysis, and drafted and finalized the manuscript. SK observed and photographed GISH slides, and provided the guidance and lab facilities for cytological studies and genomic *in situ* hybridization (GISH). DY provided the facility and guidance during phenotyping, and performed the manuscript editing. All authors contributed to the article and approved the submitted version.

## Conflict of Interest

The authors declare that the research was conducted in the absence of any commercial or financial relationships that could be construed as a potential conflict of interest.
